# International Perspectives on Pediatric Split‐Liver Allocation: A Review of Policy Frameworks and the Swiss Experience

**DOI:** 10.1111/petr.70393

**Published:** 2026-07-05

**Authors:** Barbara E. Wildhaber, Mirjam Maurer

**Affiliations:** ^1^ Department of Pediatrics, Gynecology, and Obstetrics, Swiss Pediatric Liver Center University Hospitals of Geneva Geneva Switzerland; ^2^ Unit of Pediatric Surgery, Division of Pediatric and Adolescent Surgery, Department of Pediatrics, Gynecology, and Obstetrics, University Center of Pediatric Surgery of Western Switzerland University Hospitals of Geneva Geneva Switzerland; ^3^ Swisstransplant, The Swiss National Foundation for Organ Donation and Transplantation Bern Switzerland

**Keywords:** allocation system, children, international comparison, liver transplantation, policy, regulation, split‐liver transplantation

## Abstract

Pediatric liver allocation systems vary significantly across countries, reflecting different approaches to balancing urgency, equity, and the utilization of split‐liver transplantation. This review examines international allocation frameworks and describes the evolution and impact of pediatric liver allocation policies in Switzerland. We conducted a comparative analysis of pediatric liver allocation systems in eight countries/regions (Switzerland, France, Eurotransplant, Italy, United States, United Kingdom, Canada, and Australia/New Zealand). For Switzerland, we analyzed waitlist data from the Swiss Organ Allocation System database for patients 0–16 years upon listing, comparing Era 1 (07.2007–06.2013) and Era 2 (07.2013–12.2023). International allocation systems demonstrate not only substantial variability in their approaches to pediatric priority and scoring systems, but also on split‐liver mandates. Systems range from mandatory splitting policies (Switzerland, France, United Kingdom, Italy) to encouraged approaches (Eurotransplant, United States), to discretionary approaches (Canada, Australia/New Zealand). Studies in adult recipients show that splitting does not increase complication rates. In Switzerland, the implementation of mandatory splitting for donors < 50 years with recipients ≤ 25 kg significantly reduced median waitlist times for small children from 71 days (Era 1) to 29 days (Era 2). Pediatric liver allocation systems reflect diverse national priorities and institutional capacities. Mandatory split‐liver policies can effectively reduce waitlist times for small children—without compromising the safety of adult recipients. The Swiss experience demonstrates that targeted regulatory changes, supported by advocacy and evidence, can meaningfully improve outcomes for pediatric recipients.

**Abbreviations**
IQR: interquartile range
LT: liver transplant
MELD: model for end‐stage liver disease
PELD: pediatric end‐stage liver disease

## 1 Introduction

Pediatric liver transplantation (LT) has evolved dramatically over the past four decades, transforming from an experimental procedure to a life‐saving therapy for children with end‐stage liver disease. However, the persistent shortage of suitable donor organs continues to pose significant challenges, particularly for small children who require appropriately sized grafts. This scarcity has driven the development of diverse allocation systems worldwide, each attempting to balance competing principles of medical urgency, equity, and efficient organ utilization for children and adults alike.

Split‐LT, in which a single deceased donor liver is divided to benefit two recipients, has emerged as a powerful tool to address organ scarcity [1]. By allowing one adult‐sized liver to serve both a pediatric recipient (typically receiving the left lateral segment) and an adult recipient (receiving the extended right lobe), split‐LT effectively expands the donor pool without increasing the number of deceased donors. Despite its potential, the utilization of split‐LT varies considerably across countries, influenced by technical capabilities, institutional practices, and—critically—allocation policies.

As documented by Fischler et al. [2], there are many different allocation policies for pediatric LT across the world, each reflecting distinct national priorities and healthcare system structures. This review provides a comprehensive comparison of pediatric liver allocation systems across eight major transplant regions, examining how different policy frameworks approach pediatric priority, scoring systems, and focused on split‐liver utilization. We then present the Swiss experience in detail, describing the evolution of allocation regulations over two decades and the measurable impact of policy changes on waitlist outcomes for children.

## 2 International Comparison of Pediatric (Split‐)Liver Allocation Systems

Pediatric liver allocation systems worldwide show diverse approaches to prioritizing children while maintaining equity for adult candidates. Table 1 provides a structured comparison of allocation frameworks across eight countries/regions (Switzerland, France, Eurotransplant, Italy, United States, United Kingdom, Canada, and Australia/New Zealand), showing key differences in pediatric definitions, scoring systems, and allocation hierarchies. The international comparison reveals that all systems prioritize super‐urgent patients and employ some form of severity scoring but still are very different from each other, with varying levels of urgency. Table 2 shows the comparison of pediatric split‐liver allocation in the selected countries. Substantial variation exists in the structural integration of split‐LT and additional pediatric prioritization mechanisms. Countries with mandatory splitting policies (Switzerland, France, United Kingdom, and Italy) have established split‐LT as a regulatory requirement rather than a clinical option, whereas systems with optional approaches (encouraged or discretionary) rely more heavily on center expertise and individual decision‐making. Findings are detailed in the following sections.

**TABLE 1:** Comparison of pediatric allocation frameworks.

Domain	Switzerland [3]	France [4]	Eurotransplant^a^ [5]	Italy [6]	United States [7]	United Kingdom [8]	Canada [9]	Australia/New Zealand [10]
Pediatric definition	< 18 years	< 18 years	< 18 years	< 18 years	< 18 years	< 17 years	< 18 years	< 18 years
Scoring system	MELD (with automatic updates) + urgent status^b^	MELD (with exception points upon review board decision) + super‐urgent status^b^	MELD (with automatic updates) + urgent statuses^b^	Status 3 children are listed by PELD (< 12 yo) or MELD (≥ 12 yo), then by body weight (≤ 25 or > 25 kg) and then by waiting time^c^ + urgency statuses 1, 1B, 2A, 2B, with definitions for each	PELD (< 12 yo), MELD (≥ 12 yo); statuses 1A (high super‐urgency)/1B (super‐urgency)^b^	Benefit‐based system with super‐urgency schemes and categorical pediatric priority pathways	PELD (< 12 yo), MELD (≥ 12 yo)	PELD
Allocation hierarchies	Urgency first, then MELD and waiting time; pediatric donors to pediatric recipient	Super‐urgency first, then MELD and waiting time; pediatric donors to pediatric recipient	High‐urgency first, then MELD; pediatric donors to pediatric recipient	Status 1 adults/pediatrics first; pediatric donors to pediatric recipient	Status 1A/1B first, then MELD/PELD score; pediatric donors to pediatric recipient	National tiered sequence: super‐urgent first, then prioritized pediatric indications and pediatric/small‐adult size, then elective offers ranked by Transplant Benefit Score [11]	Sickest first (PELD, MELD)	Urgent‐listed patient first on a binational basis; then ABO‐identical/compatible recipient, then PELD score

Abbreviation: yo, years old.

^a^ Austria, Belgium, Croatia, Germany, Hungary, Luxembourg, Netherlands, Slovenia.

^b^ Super‐urgent status clearly defined.

^c^ Exceptions can be made as per clear definitions, and if not transplanted within 3 months, patients pass into Status 2B.

**TABLE 2:** Comparison of pediatric split‐liver allocation.

Domain	Switzerland [3]	France [4]	Eurotransplant^a^ [5]	Italy [6]	United States [7]	United Kingdom [8]	Canada [9]	Australia/New Zealand [10]
Additional pediatric split‐regulation	Mandatory split for donors < 50 yo and if no urgency: priority goes to recipient body weight ≤ 25 kg	Mandatory split for donors < 30 yo and if no super‐urgency	No formal split‐regulation, pediatric protection mainly via pediatric MELD (automatic upgrades)	Mandatory split for pediatric donors ≥ 40 kg and/or donors ≤ 50 yo and if no super‐urgency	No formal split‐regulation, pediatric prioritization via status/score and national sharing	Structured split/reduced‐size pathways: when donor meets split criteria, left lateral segment is prioritized for pediatric/small adults. Children < 2 yo with incompatible blood group = super‐urgent	No formal split‐regulation	No formal split‐regulation
Split/partial graft mechanics	Split‐liver allocation automatically if donor < 50 yo and recipient ≤ 25 kg, unless overridden by urgency	Split‐liver allocation automatically if donor < 30 yo, and must be split, unless overridden by super‐urgency	Split‐liver transplantation is embedded in allocation sequences encouraging split if the 50/50‐rule is fulfilled	Split‐liver allocation automatically if pediatric donor ≥ 40 kg and/or donor ≤ 50 yo, unless overridden by super‐urgency	Donor liver flagged for split if donor < 40 yo, BMI ≤ 28, low vasopressors; remaining segment must be offered; match run identifies donors with split potential, centers expected to make reasonable efforts to allocate the remaining segment when split done	Split‐liver allocation automatically, if formal split‐liver criteria fulfilled: DBD donors < 45 yo, BMI < 30 kg/m^2^, acceptable liver enzymes. Left lateral segment preferentially allocated to pediatric/small adult recipients	Split‐liver transplantation remains program/center dependent	Split‐liver transplantation is discretionary and based on donor suitability, recipient needs, and center expertise
Split‐liver allocation (if conditions met)	Mandatory	Mandatory	Encouraged	Mandatory	Encouraged	Mandatory	Discretionary	Discretionary

Abbreviations: BMI, body mass index; DBD, donation after brain death; yo, years old.

^a^ Austria, Belgium, Croatia, Germany, Hungary, Luxembourg, Netherlands, Slovenia.

### 2.1 Pediatric Definitions and Age Cutoffs

Most jurisdictions define pediatric recipients as those younger than 18 years, with Switzerland stratifying into < 12 years and 12 to < 18 years categories. The United Kingdom uses < 17 years as its pediatric cutoff. These age distinctions influence scoring system selection, with Italy, the United States, Canada, and Australia/New Zealand (but not Switzerland, France, Eurotransplant, and differently, the United Kingdom) employing the Pediatric End‐Stage Liver Disease (PELD) score for younger children (< 12 years) if not all, and the Model for End‐Stage Liver Disease (MELD) score for adolescents and adults.

### 2.2 Scoring Systems, Priority Schemes, and Allocation Hierarchy

Even though the development of MELD and PELD scores represented a major advancement in liver allocation, providing objective, laboratory‐based measures of disease severity, it is known today, that neither MELD nor PELD scores accurately reflect the risk of death in pediatric patients [12, 13]. Still, all reviewed systems employ the disease severity scoring PELD or MELD, but the structure and implementation vary considerably, including categories of urgency: Switzerland and France use MELD scores with clearly defined urgent status categories. Swiss patients in urgent status have also access to the liver waitlist in France if they meet the French super‐urgent criteria. Eurotransplant similarly employs MELD scoring with urgent categories but serves a multicountry alliance (Austria, Belgium, Croatia, Germany, Hungary, Luxembourg, Netherlands, and Slovenia). Italy uses a much more comprehensive, stratified status system (1, 1B, 2A, 2B, 3) with specific definitions for each urgency level. In category 3 children are not only listed as for their PELD/MELD‐score, but also in regards of their body weight (≤ 25 or > 25 kg). The United States employs PELD for children < 12 years and MELD for older recipients. However, Status 1A (high super‐urgency) and 1B (super‐urgency) categories take precedence over MELD/PELD scores. Most different from the other countries, the United Kingdom has recently implemented a benefit‐based system using *Transplant Benefit Scores* [11], representing a paradigm shift from traditional urgency‐based allocation. This system includes super‐urgency pathways and categorical pediatric priority mechanisms. Canada and Australia/New Zealand maintain sickest‐first approaches using PELD/MELD scores with super‐urgent categories.

A consistent pattern across all systems is the prioritization of patients in urgent status as the first decision gate. Another unifying feature across systems is the pediatric donor preference: policies preferentially allocate pediatric donor livers to pediatric recipients. This practice recognizes both the size‐matching advantages and ethical considerations of prioritizing children for organs from child donors. However, systems diverge in their subsequent allocation priorities.

### 2.3 Split‐Liver Transplantation Policies

The most striking differences among allocation systems concern split‐liver policies, which can be categorized into three broad approaches.

#### 2.3.1 Mandatory Splitting With Specific Donor Criteria

Switzerland mandates splitting for donors of < 50 years when a recipient of a body weight of ≤ 25 kg is available, unless overridden by urgent status. France requires splitting for donors < 30 years, with thus a younger age cutoff than Switzerland, unless super‐urgency takes precedence. Similarly to Switzerland, Italy orders splitting for adult donors ≤ 50 years, but also for pediatric donors ≥ 40 kg, unless super‐urgency applies. The United Kingdom mandates splitting for donors meeting specific criteria such as age < 45 years, BMI < 30 kg/m^2^, and acceptable liver enzymes. This policy represents one of the most insistent split mandates globally.

#### 2.3.2 Procedurally Encouraged but Nonmandatory Splitting

In the United States, donors who have split potential (age < 40 years, BMI ≤ 28, minimal vasopressor support) are identified and flagged in the allocation system. Yet splitting is not mandatory. If splitting is decided on, centers are expected to make “reasonable” efforts to allocate the remaining segment. Eurotransplant embeds split‐LT in allocation sequences encouraging split if the “50/50‐rule” is fulfilled but does not frame it as mandatory, leaving the final decisions to transplant programs. This means that a donor liver who meets the conditions of ≥ 50 kg body weight and ≤ 50 years of age is considered a potential split‐liver donor.

#### 2.3.3 Discretionary and Center‐Dependent Nonmandatory Splitting

Canada and Australia/New Zealand leave split‐LT to center discretion based on donor suitability, recipient needs, and institutional expertise. These systems prioritize urgent patients first, with split decisions made case‐by‐case rather than through regulatory mandate.

### 2.4 Additional Pediatric Priority Mechanisms

Beyond split‐liver policies, some systems provide additional mechanisms to prioritize pediatric recipients. The United Kingdom incorporates structured split/reduced‐size pathways with automatic prioritization for pediatric/small‐adult recipients. Notably, recipients < 2 years listed in urgent status with ABO‐incompatible blood groups not only mandate splitting for this vulnerable population, but also automatically qualify for access to incompatible blood groups, given the evidence that young recipients can safely receive an ABO‐“incompatible” graft without compromising outcomes for the graft or child [14]. Eurotransplant, which has no mandatory splitting, relies primarily on automatic MELD/PELD upgrades for pediatric patients rather than explicit split mandates—a mechanism which is also practiced in Switzerland and France. In the United States, pediatric prioritization is fostered mainly through status/score mechanisms and national sharing requirements rather than mandatory splitting.

### 2.5 Organ Sharing Between Countries

To increase the donor pool for patients there are a number of well‐established cross‐border organ exchange organizations in Europe, such as *Scandiatransplant* or *Eurotransplant*. This collaborative network of eight European countries also aims to optimize the allocation of donated organs based on medical urgency, compatibility, and other criteria, regardless of national borders. Switzerland is not part of *Eurotransplant* but is a member of the *South Alliance of Transplantation*, an association of countries including France, Italy, Spain, Portugal, and Czech Republic that promotes cross‐border exchange of organs. Today, 25 European countries offer organs which cannot be allocated to a recipient within their country via the Facilitating Exchange of Organs Donated in EU Member State (FOEDUS) IT platform [15]. This exchange is extremely important, especially for pediatric recipients. International agreements have the potential to increase organ transplantation rates, an approach which may be worthwhile beyond Europe. Australia and New Zealand adopted an even more integrated approach: from the inception of their transplant programs in 1981, the two countries established a completely unified system to promote equitable access to transplantation services.

## 3 The Swiss Experience: Evolution and Impact of Allocation Reforms

### 3.1 Historical Context and Regulatory Evolution

Since the first pediatric liver transplant in 1989 at the University Hospitals of Geneva—now the national reference center for pediatric liver transplantation—Switzerland lacked a unified allocation system until 2007. This decentralized approach fostered inter‐center competition and limited transparency. The modern era of Swiss liver allocation began with the implementation of legally binding ordinances in 2007, establishing a unified national waitlist for both children and adults based on MELD scores. Already this initial system recognized special considerations for pediatric patients: pediatric donor livers (< 18 years) primarily had to be allocated to pediatric recipients if no life‐threatening urgency existed. However, an adjustment was already made in 2008, so that the National Allocation Office determined the number of MELD points for children under 12 years, after consultation with the expert group on a case‐by‐case basis (Exception MELD). This aimed to ensure that children, who often may not present with high creatinine, bilirubin or INR, are not disadvantaged by the scoring system. In 2013, the landmark “≤ 25 kg regulation” was implemented, mandating a liver split for donors < 50 years whenever a recipient ≤ 25 kg is available, unless overridden by urgency. This regulation aimed to address the specific disadvantages faced by small children on the mixed adult‐pediatric waitlist. In 2015, the “exception MELD regulation” for children was revised. Children < 12 years now receive 14 MELD points at listing, with automatic escalation of 1.5 points every 30 days, providing score‐based priority in addition to the weight‐based splitting mandate.

These regulatory changes were achieved through persistent advocacy by pediatricians and pediatric surgeons, supported by data demonstrating the disadvantages children faced on the mixed waitlist. They repeatedly advocated that children waited too long for their organ, clinically declined more rapidly than adults due to their frailty, and that liver‐sick children were no more able to thrive and grow on the waitlist and often presented with severe complications due to their liver disease, leading to life‐long sequelae, reducing their successful integration into society once adult [16]. More, a sicker child at LT consumes doubtlessly more health‐related costs than a patient in acceptable or even good condition. Further, all liver splitting for children transplanted in Switzerland is exclusively performed in situ, both in Switzerland and for the donors in France and beyond. This technique prevents prolonged cold ischemia time and therefore served as a further argument to convince physicians and surgeons treating adult patients to agree to accept the right tri‐segments instead of the whole liver. In short, the openness of colleagues treating adults, *Swisstransplant's* willingness to include pediatricians in policy discussions, and the generation of scientific data supporting pediatric needs [17], all contributed to successful regulation and policy reforms, an ongoing process still of today.

### 3.2 Methods of the Swiss Analysis

We conducted a comprehensive review of waitlist data from the Swiss Organ Allocation System (SOAS) database, including all patients 0–16 years listed for from July 1, 2007, to December 31, 2023. Patient inclusion was limited to those under 16 years of age, in accordance with the Swiss legal definition of a pediatric recipient. The analysis compared two eras: Era 1 (July 2007–June 2013, pre‐≤ 25 kg regulation) and Era 2 (July 2013–December 2023, post‐≤ 25 kg regulation).

Data on pediatric LT, including graft type and geographic origin, were analyzed for both eras. We performed analysis of the entire 0–16 years cohort and separately analyzed the subgroup of children ≤ 25 kg at LT. No ethical approval was required as data were extracted anonymously from the SOAS database with appropriate consent for data use. Given the small cohort size, we only performed basic statistical analysis using the Wilcoxon rank‐sum test for individual parameters in this primarily descriptive analysis.

For contextual information, numbers of transplanted patients and types of liver grafts during Era 1 and Era 2 are summarized in Table 3a for children and Table 3b for adults.

**TABLE 3:** Numbers of transplanted patients (a: children, b: adults) and types of liver grafts during Era 1 and 2.

(a) Children				
	Era 1 (7/2007–6/2013)		Era 2 (7/2013–12/2023)	
	All children	Children ≤ 25 kg	All children	Children ≤ 25 kg
On liver waitlist	57	45	96	62
Out of waitlist	52	44	89	59
Transplanted < 16 years old	47 (7.8/year)	41 (6.8/year)	86 (8.2/year)	57 (5.4/year)
> 16 years old at LT	1	0	4	0
Death on waitlist	1 (1.8%)	1 (2.2%)	2 (2.1%)	2 (3.2%)
Remained on waitlist	4	1	3	3
Graft type				
Left lateral segment	30	29	51	47
Right lobe graft	0	0	0	0
Right tri‐segment	2	0	4	0
Whole	15	12	31	10

(b) Adults				
	Era 1 (7/2007–6/2013)		Era 2 (7/2013–12/2023)	
On liver waitlist	977		2231	
Out of waitlist	883		1967	
Transplanted	578 (96/year)		1359 (129.4/year)	
Death on waitlist	141 (14.4%)		330 (14.8%)	
Remained on waitlist	114^a^		277^a^	
Graft type				
Left lateral segment	6		9	
Right lobe graft	26		32	
Right tri‐segment	16		28	
Whole	530		1290	

^a^ Twenty and thirteen patients, respectively, were most probably added secondarily to the liver waitlist after having initially been listed for another organ.

### 3.3 Impact on Waitlist Times and Outcomes

The most dramatic impact of the 2013 regulatory changes was observed in waitlist times for small children ≤ 25 kg at LT, this upon exclusion of children in urgent status or having received a living donor graft, since they did not rely on the ≤ 25 kg regulation. Median waiting time for these children significantly decreased from 71 days (IQR 38–107) in Era 1 to 29 days (IQR 19–67) in Era 2 (*p* = 0.037), representing an approximately 60% reduction. For all children < 16 years, median waiting time decreased nonsignificantly from 58 days (IQR 38–106) to 45 days (IQR 21–193) (*p* = 0.92), excluding patients receiving living donor grafts or listed as urgent.

Importantly, waitlist mortality remained stable and low throughout both eras. Overall waitlist mortality was 2% for all children and 2.8% for children ≤ 25 kg, with no significant differences between eras. The three children who died on the waitlist during the study period all died within 1–3 days of listing, suggesting acute presentations rather than prolonged waitlist deterioration.

### 3.4 Changes in Graft Type and Origin

The implementation of mandatory splitting led to measurable changes in graft type distribution. For small children ≤ 25 kg, the proportion receiving left lateral segments increased from 71% in Era 1 to 82% in Era 2. This around 10% increase of grafts being left lateral segments from Swiss donors (notably despite a decrease in young donors) shows that split‐liver use is an effective strategy to reduce time on waitlist for small children.

Geographic origin of grafts shifted notably during the study period. As mentioned above, Switzerland maintains a close collaboration with France, allowing organ exchange in urgent situations or when suitable local recipients are unavailable. France has higher donor rates (25–30 per million population) compared to Switzerland (17–19 per million population) [18], making this partnership crucial for Swiss transplant programs. Our data show evolving utilization of this cross‐border cooperation, with French grafts playing an important role, particularly for urgent listings. Interestingly, even for pediatric waitlist patients not requiring urgent status, grafts often derived from France or other European countries, showing that organ sharing in Europe is common.

### 3.5 Urgent Status Utilization

Interestingly, both Switzerland and, as of our knowledge, also France, experienced increasingly liberal use of urgent status for children over the study period. The proportion of children transplanted in urgent status increased from 32% in Era 1 to 37% in Era 2 for all children < 16 years, and from 29% to 37% for small children ≤ 25 kg. Indeed, there was an overall increase in urgent status of 8% between Era 1 and 2 for all but tumor‐patients < 16 years old (tumor‐patients were excluded from this analysis since automatically in urgent status upon listing). Yet, for the small children, there was only an increase of 4% between Era 1 and 2. These results suggest that, despite a generally more widespread use of urgent status in Era 2, there seems to be a reduced necessity for its use in recipients weighing ≤ 25 kg. This suggests that the new legislation, that is the “≤ 25 kg regulation,” had a favorable influence on the need for urgent status. This is supported by the finding that in Era 2, there was a reduced need for *secondary* urgent listing of small children after spending several weeks on the standard waitlist. The new regulation therefore appears to have led to a reduction in urgent status listings owing to clinical deterioration of small children.

## 4 Discussion

### 4.1 The Case for Mandatory Split‐Liver Transplantation

Split‐LT represents a highly effective strategy for addressing organ scarcity and decreasing waitlist mortality, particularly for pediatric and size‐constrained recipients. Yet, despite widespread technical capability to perform split procedures, utilization rates vary dramatically based on policy frameworks rather than surgical expertise. The Swiss experience presented in this paper provides evidence for the benefits of mandatory splitting policies. This finding is supported by data from other countries with regulatory mandates such as Italy [19, 20]. Further, US data clearly show that increased utilization of split‐LT could substantially reduce pediatric waitlist mortality [21]. Indeed, in nonmandatory allocation systems such as the United States pediatric waitlist mortality remains a persistent challenge, with rates as high as 5.9%, and even higher for children under two [22]. In Eurotransplant, for children with biliary atresia (the most common indication for pediatric LT) waitlist mortality is as high as 7.9% [23].

Despite clear benefits, split‐LT has historically met with reluctance, largely due to concerns about increased technical complexity, prolonged operative times, and potential for inferior outcomes. While these concerns were valid in earlier eras, contemporary evidence does not support them as justifications for continued underutilization. Many studies support equivalent outcomes for split‐LT and whole LT when appropriate donor selection criteria are applied, with comparable patient and graft survival, as well as no difference in early complication rates [17, 24, 25]. Further, it has been demonstrated, in pediatric and adult recipients, that there is a survival advantage also for adult patients associated with accepting a splittable graft rather than waiting for a subsequent whole liver offer: United States registry data showed that accepting a split liver offer was associated with a significant 43% reduction in mortality compared to declining and waiting for a subsequent offer, with 92.2% versus 84.4% 1‐year survival after the decision [26]. Modern surgical techniques, improved preservation methods, and accurate recipient selection have largely eliminated the historical survival disadvantages associated with split grafts [27].

Beyond individual patient outcomes, mandatory splitting provides systemic benefits by effectively doubling the utility of suitable donor livers. Each split procedure creates two functional grafts where previously only one recipient would benefit, expanding the donor pool without requiring increased deceased donor numbers. This efficiency gain is particularly valuable given persistently low organ donation rates in many countries.

Broader implementation of split‐LT may also address persistent disparities in transplant access. Smaller recipients, including infants, young children, and small adults (often women), have historically faced disadvantages on mixed waitlists dominated by larger adult candidates. Weight‐based priority mechanisms, as implemented in Switzerland, France, Italy, and the United Kingdom, directly address this structural inequity by ensuring that appropriately sized grafts reach those who need them most.

It seems that the persistent gap between technical capability and actual utilization in many jurisdictions suggests that policy frameworks—rather than clinical limitations—represent the primary barrier to expanded split‐LT.

### 4.2 International Context and Variability

The international comparison presented in this review reveals substantial heterogeneity in approaches to pediatric liver allocation. While all systems recognize the need to prioritize children in some contexts, the specific mechanisms employed vary considerably. Countries with mandatory splitting policies (Switzerland, France, United Kingdom, and Italy) have institutionalized split‐LT as a regulatory requirement, whereas others (United States and Eurotransplant) encourage but do not mandate splitting, and still others (Canada and Australia/New Zealand) leave decisions entirely to center discretion.

This variability likely reflects differences in national contexts, including organ availability, geographic factors, institutional capabilities, and cultural attitudes toward regulatory intervention in clinical practice. Despite high waitlist mortality rates that clearly reflect the severity of organ shortage, the United States has to date not implemented comprehensive allocation reforms, which certainly must be related to other reasons. Notably, several reports have highlighted the persistently poor utilization of split‐liver transplantation in the United States [28, 29].

Geographic factors also matter: compact countries or regions may consider centralized splitting more feasible than those with vast distances between transplant centers.

The United Kingdom's recent shift in 2018 to a benefit‐based allocation system represents an intriguing paradigm departure from traditional urgency‐based models. The National Liver Offering Scheme (NLOS) offers deceased donor livers based on calculated transplant benefit for most patients. This benefit calculation incorporates both patient need and predicted posttransplant utility. The scheme has demonstrated reduced waitlist mortality while preserving posttransplant outcomes, thereby maximizing life‐year gain across the waiting list population [11]. Whether this model proves superior in small pediatric patients remains to be evaluated.

### 4.3 Lessons From the Swiss Experience

The Swiss data demonstrate that targeted regulatory interventions can meaningfully improve pediatric LT access without compromising safety for either adult or pediatric recipients. The significant reduction in median waitlist times for small children following the 2013 reforms represents a clinically significant improvement, allowing children to receive transplants in better overall condition and potentially avoiding complications associated with prolonged waitlist times. Our experience also highlights the importance of advocacy in driving policy change. Persistent efforts by pediatric specialists to document disadvantages faced by children, combined with a willingness from adult transplant colleagues and regulatory bodies to consider evidence‐based reforms, created conditions for meaningful improvement.

This said, we must mention that emerging perspectives in pediatric LT include not only policy changes, but that (i) living donor transplantation which has been shown to have superior long‐term patient and graft survival compared to deceased donor LT, (ii) artificial intelligence‐assisted allocation models that seem to significantly outperform traditional MELD‐based scoring systems in predicting waitlist mortality and could reduce annual deaths, and (iii) machine perfusion technologies have shown to reduce early allograft dysfunction and biliary complications, and improve 1‐year graft survival compared to static cold storage [30, 31, 32].

### 4.4 Limitations

The international comparison relies on policy documents rather than outcome data, preventing direct assessment of which allocation models produce optimal results. Future research, despite being challenging, should employ comparative effectiveness methods to evaluate outcomes across different allocation frameworks, ideally using international detailed registry data.

Additionally, this review focuses primarily on waitlist outcomes (waiting times, mortality) rather than posttransplant results. While evidence suggests split grafts perform comparably to whole livers, ongoing surveillance of long‐term outcomes remains important.

## 5 Conclusion

International pediatric liver allocation systems demonstrate substantial variability in their approaches to prioritizing children, employing split‐LT, and clearly trying to balance competing principles of urgency and equity. While all systems recognize the need for urgent status pathways and employ some form of severity scoring, they differ markedly in whether split‐LT is mandated, encouraged, or left to center discretion.

The Swiss experience demonstrates that mandatory split‐liver policies, particularly when combined with weight‐based priority mechanisms, can significantly reduce waitlist times. This experience offers valuable lessons for other jurisdictions: meaningful policy reform is possible when grounded in data, guided by patient welfare, and advanced through persistent, collaborative advocacy.

Ultimately, the goal of any allocation system must be to ensure that all patients—pediatric and adult alike—have equitable access to life‐saving transplantation. Thoughtfully designed regulations, informed by evidence and responsive to patient needs, represent strong tools for achieving this fundamental objective.

## Funding

The authors have nothing to report.

## Conflicts of Interest

The authors declare no conflicts of interest.

## Data Availability

The data that support the findings of this study are available on request from the corresponding author. The data are not publicly available due to privacy or ethical restrictions.
